# Permittivity Measurement in Multi-Phase Heterogeneous Concrete Using Evidential Regression Deep Network and High-Frequency Electromagnetic Waves

**DOI:** 10.3390/ma18163766

**Published:** 2025-08-11

**Authors:** Zhaojun Hou, Hui Liu, Jianchuan Cheng, Qifeng Zhang, Zheng Tong

**Affiliations:** 1 School of Transportation, Southeast University, Nanjing 210018, China; 2 CCDI (Suzhou) Exploration & Design Consultant Co., Ltd., Suzhou 215007, China; 3 China Railway 14th Bureau Large Shield Engineering Co., Ltd., Nanjing 211800, China

**Keywords:** permittivity, high-frequency electromagnetic wave, multi-phase heterogeneous concrete, Gaussian random fuzzy number, uncertainty quantification

## Abstract

Permittivity measurements of concrete materials benefit from the application of high-frequency electromagnetic waves (HF-EMWs), but they still face the problem of being aleatory and exhibit epistemic uncertainty, originating from multi-phase heterogeneous materials and the limited knowledge of HF-EMW propagation. This limitation restricts the precision of non-destructive testing. This study proposes an evidential regression deep network for conducting permittivity measurements with uncertainty quantification. This method first proposes a finite-difference time-domain (FDTD) model with multi-phase heterogeneous concrete materials to simulate HF-EMW propagation in a concrete sample or structure, obtaining the HF-EMW echo that contains aleatory uncertainties owing to the limited knowledge of wave propagation. A U-net-based model is then proposed to denoise an HF-EMW, where the difference between a couple of observed and denoised HF-EMWs characterizes aleatory uncertainty owing to measurement noise. Finally, a Dempster–Shafer theory-based (DST-based) evidential regression network is proposed to compute permittivity, incorporating the quantification of two types of uncertainty using a Gaussian random fuzzy number (GRFN): a type of fuzzy set that has the characteristics of a Gaussian fuzzy number and a Gaussian random variable. An experiment with 1500 samples indicates that the proposed method measures permittivity with a mean square error of 7.50% and a permittivity uncertainty value of 74.70% in four types of concrete materials. Additionally, the proposed method can quantify the uncertainty in permittivity measurements using a GRFN-based belief measurement interval.

## 1. Introduction

Permittivity, a key parameter that characterizes the electrical properties of materials, represents the ability to store electric energy in an electromagnetic field. It reflects how high-frequency electromagnetic waves propagate within the material, including aspects such as attenuation, wavelength, and speed [1]. In the field of civil engineering, particularly in the study and application of cement-based composite materials, the accurate measurement of the dielectric constant of pavement structures holds significant importance.

In practical applications, the measured permittivity is widely utilized in several critical areas. In thickness measurements, accurate permittivity values provide a reliable basis for evaluating the dimensions of structural elements, ensuring that building components meet design specifications [2]. For subsurface object detection, permittivity measurement helps accurately locate and identify objects hidden within structures, aiding in the detection of potential safety hazards [3]. In the analysis of material corrosion, monitoring changes in the permittivity can effectively evaluate the degree of corrosion [4]. For studies related to energy absorption characteristics, permittivity data serves as crucial support in the development of composites with specific absorption properties [5]. In high-throughput composite material design, permittivity measurements guide material optimization and innovation [6].

Despite its advantages, accurately measuring the permittivity of cement concretes remains a challenging task. Existing methods (e.g., the parallel plate method, probe method, resonant cavity method, waveguide method, free-space method) can generally be categorized into two groups: transmission-time-based and reflection-coefficient-based techniques [7]. These approaches are commonly affected by two types of uncertainties: aleatory uncertainty and epistemic uncertainty. Aleatory uncertainty refers to the inherent randomness property in the measurement process, such as the multi-phase heterogeneous property from the random spatial distribution of cement paste and aggregates and variability in emission and reception. Epistemic uncertainty arises from limitations in knowledge, modeling assumptions, or incomplete understanding of the physical processes involved. For instance, in transmission-based methods, the assumptions regarding wave paths and homogeneous media may not match actual complex conditions, leading to biased or inaccurate results.

### Hypothesis and Research Objectives

To solve the problem of uncertainty quantification in the permittivity measurement of concrete materials, this paper proposes an evidential regression deep network for conducting permittivity measurements with uncertainty quantification. This method has the following two hypotheses:

1. An HF-EMW is generated by a pulse-frequency antenna with a fixed frequency. This paper does not discuss the stepped-frequency antenna.
2. The finite-difference time-domain (FDTD) model simulates HF-EMW propagation in a concrete sample or structure without knowledge uncertainty since the propagation path can be completely observed in the simulation.

In general, this method first proposes a finite-difference time-domain (FDTD) model with multi-phase heterogeneous concrete materials to simulate HF-EMW propagation in a concrete sample or structure, obtaining the HF-EMW echo that contains aleatory uncertainty owing to the limited knowledge of wave propagation. A U-net-based model is then proposed to denoise HF-EMW, where the difference between a couple of observed and denoised HF-EMWs characterizes aleatory uncertainty owing to measurement noise. Finally, a Dempster–Shafer theory-based (DST-based) evidential regression network is proposed to compute permittivity, incorporating the quantification of two types of uncertainties using a Gaussian random fuzzy number (GRFN). The main contribution of this study can be summarized as follows:

1. The proposed method can measure permittivity in four types of multi-phase heterogeneous concrete materials, exceeding other SOTA methods in this task. The proposed method is also stable when used on the four concrete types. Thus, a user can directly use the method to measure the permittivity of concrete materials using recorded HF-EMWs.
2. The proposed method can quantify the uncertainty in permittivity measurement using a GRFN-based BMI, demonstrating that the proposed method can quantify and remove the effects of aleatory and epistemic uncertainty in the permittivity measurement. A GRFN in the proposed method uses μ(x), σ(x), and h(x) as the most plausible permittivity value in the measurement process and the variability around these values as aleatory uncertainty and epistemic uncertainty, respectively.
3. The superiority of the proposed method benefits from the FDTD simulation and the denoising deep network. The FDTD simulation quantifies epistemic uncertainty due to limited knowledge, such as HF-EMW propagation. The denoising deep network quantifies aleatory uncertainty owing to the measurement noise, such as the multi-phase heterogeneous property of concrete materials.

## 2. Related Work

### 2.1. Permittivity Characterization with High-Frequency Electromagnetic Waves

Permittivity is a fundamental electrical property that describes how materials interact with electric fields. At high frequencies ranging from megahertz (MHz) to terahertz (THz), the characterization of permittivity becomes increasingly significant, particularly in applications involving wireless communication [8], radar systems [9], geophysical exploration [10], and biomedical diagnostics [11]. Complex permittivity encapsulates both energy storage and dielectric losses within a material. As the frequency increases, dispersive and relaxation phenomena, such as dipole polarization, ionic conduction, and interfacial effects, become more pronounced, requiring accurate and frequency-resolved measurement techniques.

Multiple high-frequency measurement techniques have been developed to characterize permittivity, as summarized in Table 1. Among the most used are the vector network analyzer-based transmission/reflection methods [12]. These rely on measuring the scattering parameters of a sample inserted in a coaxial line, waveguide, or free-space setup. Coaxial probes [13], particularly the open-ended type, are widely used due to their ease of use and suitability for soft or liquid materials. Waveguide-based methods [14] offer higher accuracy but require more elaborate sample preparation. Free-space measurements, on the other hand, allow non-contact and broadband evaluation of flat samples, making them ideal for high-frequency applications in the GHz to THz range.

Resonant cavity methods provide extremely accurate permittivity measurements at discrete frequencies by exploiting the shift in resonant frequency and quality factor that occurs when a sample is inserted. These techniques are particularly suitable for low-loss dielectric materials. In contrast, time-domain methods like time-domain reflectometry [15] and terahertz time-domain spectroscopy [16] provide broadband information. THz-TDS has emerged as a powerful tool for characterizing permittivity in the sub-millimeter wave regime, offering femtosecond time resolutions and high-frequency bandwidths. This method is especially effective for polymeric materials, where molecular vibrations and carrier dynamics can be captured.

Recent advances integrate time-domain methods and deep learning-based algorithms with inverse electromagnetic modeling to improve the robustness and efficiency of permittivity extraction [17,18]. These data-driven methods are particularly useful in heterogeneous or layered media where traditional inversion becomes unstable. Additionally, the rise of metamaterials and complex engineered structures has necessitated new characterization approaches that account for anisotropy, dispersion, and nonlinearity. Researchers have also developed high-temperature and in situ permittivity measurement systems to support material evaluation in harsh environments, such as reactors, engines, and planetary exploration.

Despite significant progress, several challenges remain. Calibration procedures at high frequencies are sensitive to measurement artifacts, and uncertainties in sample geometry or positioning can significantly affect results. There is also a trade-off between spatial resolution and frequency coverage: techniques with high-frequency resolutions often sacrifice spatial detail and vice versa. Furthermore, interpreting permittivity data from inverse problems remains complex, particularly when measurements are sparse or noisy.

In summary, permittivity characterization using high-frequency electromagnetic waves is a mature but still rapidly evolving field. It plays a critical role in modern science and engineering, from designing communication systems to diagnosing diseases and understanding materials at the molecular level. Ongoing advancements in sensor miniaturization, computational electromagnetics, and AI-driven analysis are likely to enhance further the precision, accessibility, and real-time capabilities of high-frequency permittivity measurements.

### 2.2. Uncertainty Quantification in Material Permittivity Measurement

Uncertainty analysis refers to the process of studying, quantifying, and evaluating the various uncertainties inherent in measurement procedures. In the context of ground-penetrating radar (GPR) applications, such as permittivity measurement [19] and signal processing [20], uncertainties are pervasive and significantly affect the reliability and accuracy of the results. From the perspective of their nature of origin, uncertainties are typically classified into aleatory uncertainty and epistemic uncertainty [17]. Theoretically, aleatory uncertainty is associated with intrinsic random fluctuations in the system. In GPR-based measurements, this is most evident in the form of electronic noise from the radar equipment and unpredictable electromagnetic interference from the surrounding environment. These disturbances are inherently stochastic and cannot be eliminated simply by refining measurement procedures or acquiring more domain knowledge.

On the other hand, epistemic uncertainty [21] arises from an incomplete or imprecise understanding of the system or the environment being measured. In GPR applications, epistemic uncertainty may result from limited knowledge of the complex subsurface medium or the simplifications and approximations inherent in the adopted signal processing models. For example, assumptions regarding electromagnetic wave propagation paths in heterogeneous media or the use of idealized dielectric models contribute to such uncertainty. Unlike aleatory uncertainty, epistemic uncertainty can be gradually reduced as understanding of the measurement process and the physical system improves through theoretical advancements or empirical investigations.

Over the years, numerous approaches have been proposed to quantify and model uncertainty. Early methods primarily relied on classical mathematical frameworks, such as Bayesian probability theory and Monte Carlo simulations, to estimate the probability of uncertain events. With the advent of computational methods, more sophisticated models were introduced, including interactive fuzzy sets [22], parametric and non-parametric probability density models [23], and fuzzy entropy measures [24]. These approaches utilize fuzzy set theory to represent uncertainty in multi-attribute decision-making problems, where interdependencies among attributes are prevalent. Fuzzy relations are constructed to quantify such interactions, and membership functions are employed to describe the degree to which an object belongs to different classes, thereby allowing uncertainty in classification to be quantified. Additionally, many practical models assume that uncertainty follows a specific probability distribution (e.g., Gaussian or normal distributions), where probability density functions are estimated from historical or experimental data.

In recent years, the integration of uncertainty theory with deep neural networks has attracted growing attention. For instance, Denœux et al. (2021) [25] proposed an evidence-based classifier combining Dempster–Shafer (DS) theory with deep learning. In this framework, deep learning is first used to extract features from data, which are then interpreted as evidence in DS theory. Belief and plausibility functions are computed to represent uncertainty in interval form. Tong et al. [26,27] applied an evidence transformer for pavement defect segmentation, which effectively captured complex spatial features. By incorporating DS theory, the model was able to output predictions with embedded uncertainty information, improving the reliability of the segmentation results. More recently, in 2025, Huang et al. [28] applied deep learning networks to extract features from multimodal medical images, translating those features into DS-theoretic evidence. Gaussian random fuzzy numbers were then used to represent both aleatory and epistemic uncertainties during measurement. These recent studies have opened new avenues for combining deep learning with formal uncertainty quantification frameworks. Importantly, they also address earlier limitations where different types of uncertainties were not clearly distinguished, thus enriching the expressiveness and interpretability of uncertainty-aware models.

Despite its advantages in modeling both aleatory and epistemic uncertainty, the application of DS theory in material sciences presents two challenges. First, DS theory requires the definition of a frame of discernment and basic belief assignments, which may be difficult to establish when dealing with continuous-valued or highly variable physical measurements such as permittivity. Second, there are still no existing methods integrating DS theory and deep neural networks to quantify the uncertainty in the time-domain methods for permittivity measurement.

## 3. Proposed Method

### 3.1. Overview

**Figure 1:**
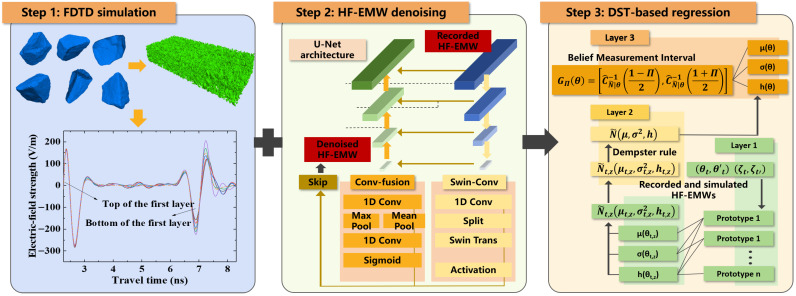
Overview of the proposed method.

This study proposes a DST-based method to characterize the permittivity distribution with uncertainty quantification in concrete materials. The main steps of this method consists of three steps, as shown in Figure 1:

**Step 1.**  A finite-difference time-domain (FDTD) model with multi-phase heterogeneous concrete materials is adopted to simulate HF-EMW propagation in a concrete sample or structure. This model obtains the HF-EMW echo, which minimizes epistemic uncertainty but still contains aleatory uncertainty. This is because the FDTD model has a well-known and calibrated wave propagation path and relationship between wave amplitude and permittivity. However, it still contains measurement noise from the random distribution of multi-phase components in a concrete sample. The FDTD model will be introduced in Section 3.2. Please note that the FDTD model was proposed in [29,30]. The contribution of this step is to use simulation results to quantify the epistemic uncertainty in permittivity measurements.
**Step 2.**  A U-Net-based model is proposed to denoise an HF-EMW signal. This model is used to obtain an HF-EMW without epistemic uncertainty, as it can remove noise derived from measurement errors, such as multi-phase heterogeneous of cement and aggregates in a concrete sample. Thus, the difference between the inputs and outputs can be the aleatory uncertainty of the permittivity measurement using an HF-EMW. The details of the U-net-based model will be described in Section 3.3.
**Step 3.**  A DST-based evidential regression network is proposed to compute permittivity with aleatory and epistemic uncertainty. The inputs of this network consist of a recorded HF-EMW from real-world measurements of a concrete sample and an HF-EMW echo from an FDTD model that simulates real-world measurements. The outputs are the permittivity of the concrete sample with the quantification of aleatory and epistemic uncertainty. The details of the DST-based evidential regression network will be introduced in Section 3.4.

### 3.2. Finite-Difference Time-Domain Simulations for Multi-Phase Concrete

#### 3.2.1. Component Distribution Simulation Using Discrete Element Method

The finite-difference time-domain (FDTD) method was employed to simulate HF-EMW propagation within a concrete sample. The simulation is conducted using GPRMax v.3.1.5, an open-source software widely used for modeling electromagnetic wave propagation. However, GPRMax’s default geometry commands only allow the construction of basic shapes such as rectangular blocks, spheres, and cylinders, limiting its ability to represent the irregular geometries of real-world pavement materials, such as coarse aggregates and cement mortar. As a result, simulations often rely on layered homogeneous models to approximate heterogeneous media, which introduces significant discrepancies between the simulation outputs and actual HF-EMWs.

To address this limitation, this study incorporates a discrete element modeling (DEM) approach, which is well-established in material mechanics for simulating complex micro-structures. Specifically, this method approximates the surface contours of arbitrarily shaped materials by randomly distributing spherical particles of varying sizes. The resulting geometric models enable accurate reconstruction of the true morphology and volume fractions of different material phases within concrete samples.

The modeling workflow begins with finite element software to construct detailed material structure models, which are then imported into GPRMax for HF-EMW propagation simulation. Compared with conventional homogeneous models, these reconstructed heterogeneous models offer significantly improved realism in capturing radar wave behavior. The PFC3D software is used to reconstruct the material distribution within concrete samples.

**Figure 2:**
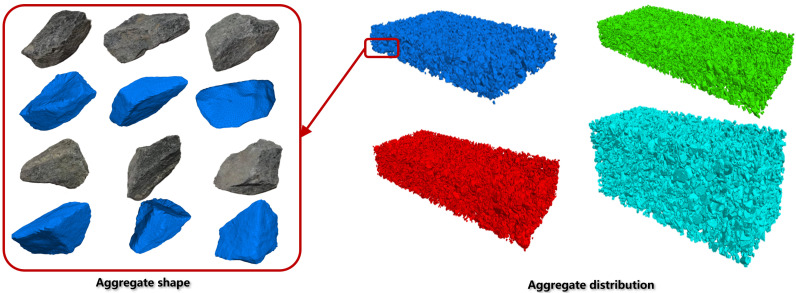
Multi-phase heterogeneous concrete model.

Real aggregate particles are first scanned using multi-view laser scanning technology to obtain dense point cloud coordinate data. Spherical fitting is then applied to approximate the surface geometry of the aggregates, enabling 3D reconstruction of aggregate phase contours. Six real aggregate particles of different sizes and shapes are selected from high-performance concrete mixtures to serve as representative samples, as shown in Figure 2. These samples are placed on an automated rotary platform and scanned using a handheld laser scanner to capture their surface geometry from all angles.

The scanned point clouds are imported into Geomagic Studio, where the surface contours of each aggregate are reconstructed. Based on the 3D point cloud data, a contour-sphere approximation method is applied. Using the Bubble Pack algorithm, multiple spheres of different radii are generated to fill the internal volume of each aggregate within the point cloud coordinate system. This process allows the geometric approximation of complex aggregate shapes and the construction of a library of 3D particle models.

The aggregates are then randomly distributed in the 3D space according to target gradation curves, forming multi-phase heterogeneous concrete samples. The samples have a size of 1.5 m × 1.5 m. The mass proportions of aggregates with different sizes are shown in Table 2.

The generated aggregate particle models were imported into PFC3D as clump templates, representing six distinct aggregate types. These clump templates were then randomly deployed within the discrete element model according to a graded distribution scheme. The placement followed a descending size order, aligning with the target gradation curve: Larger particles were placed first to form a skeletal framework, while smaller particles were subsequently filled into the voids between the larger aggregates, thereby creating a hierarchical, well-graded packing structure. For fine aggregates with particle sizes ranging from 0.6 mm to 4.75 mm, spherical particle models available in PFC3D were used due to their relatively small volume. After the skeleton and fine aggregates were placed, a vertical compressive load was applied to the model to simulate compaction and achieve a dense packing configuration. Four samples of different concrete materials are shown in Figure 2.

#### 3.2.2. FDTD Simulation

The electromagnetic simulation is performed using the GPRMax software. To incorporate the discrete element model generated by PFC3D into GPRMax, the latter’s built-in functionality is utilized to convert image data into the HDF5 format required by its electromagnetic FDTD solver. Specifically, the 3D discrete element model of the concrete sample is reconstructed, and random cross-sectional images of the heterogeneous material distribution were generated. After preprocessing, the spatial distribution and morphology information from these images were converted into HDF5 files. These files were then imported into GPRMax, where the permittivities of all component phases are defined, enabling the simulation of electromagnetic field components of HF-EMW propagating through the multi-phase heterogeneous sample. The detailed settings for the electromagnetic FDTD model in GPRMax included the sample, electromagnetic parameters, grid resolution, model dimensions, and scan duration, as described as follows.

**Model Sizes:** The size of the multi-phase heterogeneous sample is 1.5 m × 1.5 m × *D*, where *D* denotes the thickness of 0.08 m, 0.05 m, 0.08 m, and 0.05 m for Materials 1, 2, 3, and 4, respectively. Vertically, each multi-phase heterogeneous sample also consists of a 0.01 m thick air layer.

**Air-Layer Configuration:** The air layer provides space to position the antenna, maintaining a vertical separation of 0.15 m between the antenna’s center and a sample’s surface. This setup replicates the actual measurement conditions of air-coupled GPR systems. It effectively suppresses the direct ground wave interference on interface reflections from the top surface of the cement panel, thereby enhancing the distinguishability of the target reflected signals. The bottom air layer serves to eliminate boundary reflection artifacts that could otherwise interfere with the integrity and accuracy of reflection signals from the lower interface of the concrete samples.

**Antenna Frequency, Time Window, and Other Parameters:** Considering the propagation characteristics of electromagnetic waves in heterogeneous materials, as well as the trade-offs among signal resolution, penetration depth, and antenna frequency, other parameters of the electromagnetic finite-difference model were selected based on industry empirical values. These parameters are summarized in Table 3.

**Permittivity Settings:** In the model construction, void (air gap) regions are assigned around a concrete sample. By adjusting the permittivity values, different filling media conditions inside the voids can be simulated. When the void is completely dry, it is filled with air, with a relative dielectric constant of 1.00. Conversely, when fully saturated, the void is filled with water, for which its relative dielectric constant is 81.00.

For multi-phase heterogeneous media, basalt is selected as the aggregate material for particle sizes in the millimeter range, with its permittivity set to 8.00. Considering the frequency-dispersive nature of cement concrete, the permittivity of the composite phase “aggregate (mm)-cement mortar-pore” is computed as

(1) $$\varepsilon_{c}=\varepsilon_{\mathrm{avg}}=\varepsilon_{a}V_{a}+\varepsilon_{m}V_{m}+\varepsilon_{p}V_{p}$$

where $\varepsilon_{c}$ is the effective dielectric constant of the composite phase; $\varepsilon_{a}$, $\varepsilon_{m}$, and $\varepsilon_{p}$ are the dielectric constants of the aggregate, mortar, and pores, respectively; $V_{a}$, $V_{m}$, and $V_{p}$ represent their volume fractions. The aggregate particle size is denoted by the subscript *a*.

### 3.3. Deep Denoising Network

**Figure 3:**
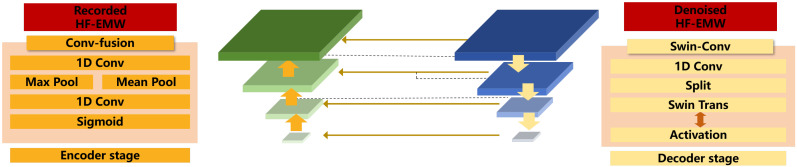
Architecture of an HF-EMW deep denoising network.

A Unet model is designed to denoise the HF-EMWs with encoder, bottleneck, and decoder modules, as shown in Figure 3. Compared with other signal denoising architectures, such as LSTM and CNN-RNN hybrids, the U-Net architecture enables efficient feature extraction across multiple temporal scales through its encoder–decoder structure and skip connections. This not only preserves fine-grained waveform details but also facilitates effective noise suppression. Additionally, the incorporation of attention mechanisms and Swin Transformer modules further enhances the U-Net’s ability to capture both local and global signal patterns, making it particularly well-suited for handling the noisy, multi-component characteristics of HF-EMW data. This advantage will be demonstrated in Section 4.3.

**(1) Encoder module**.

The encoder of the UNet architecture is designed to progressively extract features from the input one-dimensional signal through a series of downsampling operations, thereby constructing multi-scale feature representations.

The encoder consists of three cascaded downsampling modules, each composed of a residual block, an attention mechanism, and a pooling layer. The encoder takes a preprocessed and augmented input signal of shape (batch_size, 1, signal_length).

In the first downsampling module, the input signal is initially processed by a ResidualBlock1D for feature extraction. This block uses two convolutional layers with batch normalization and a skip connection, which helps alleviate the vanishing gradient problem and retains part of the original signal features. Following this, the AttentionBlock1D applies global average pooling and fully connected layers to generate channel-wise attention weights, enabling the network to enhance important features and suppress irrelevant information during training adaptively. After this stage, the number of channels expands from 1 to base_filters, while the signal length remains unchanged.

The two downsampling modules follow a similar structure but include max-pooling layers for temporal downsampling. Each pooling operation reduces the signal length by half, and the channel dimension is doubled through convolution. Specifically, the second module increases the channel dimension to base_filters × 2, and the third module further increases it to base_filters × 4, while the signal length is reduced to one-half and one-quarter of the original.

Because shallow layers use small convolution kernels with limited receptive fields, their feature maps correspond directly to local variations in the waveform, making them more effective in capturing local signal features. In contrast, deeper layers with attention mechanisms focus on low-frequency global features, as the abstracted signal representations in deeper layers are less concerned with fine-grained waveform details and more with statistical distributions. Hence, deeper modules are designed with larger receptive fields to capture the global context.

Through this hierarchical structure, the encoder combines the locality of convolution with multi-scale abstraction, preserving both fine-grained and global features of the signal. After passing through the three cascaded down-sampling modules, the encoder outputs a high-level feature representation of shape (batch_size, base_filters × 8, signal_length/8).

**(2) Swin Transformer**

Traditional CNNs rely solely on local receptive fields, which limits their ability to capture long-range dependencies in sequential signals. The introduction of a bottleneck layer addresses this issue. Specifically, the bottleneck receives the final output from the encoder, which has a shape of (batch_size, base_filters × 8, signal_length/8).

To adapt this high-level feature tensor for processing by the Swin Transformer, it is first reshaped into a two-dimensional feature map of size 20×24. The transformed feature representation is then fed into a **Swin Transformer** module. The Swin Transformer employs a *window-based self-attention mechanism*, enabling the model to capture **cross-window dependencies** and integrate the global contextual information of the signal effectively. After processing through the Swin Transformer, the output retains the same shape as the input to the bottleneck layer, i.e., (batch_size, base_filters × 8, signal_length/8).

**(3) Decoder**

The decoder mirrors the structure of the encoder and constitutes the right half of the U-shaped UNet architecture. It progressively restores the signal details through transposed convolutions (deconvolutions), skip connections, and feature fusion.

The decoder takes as input the high-level feature representation from the bottleneck layer, with a shape of [B, base_filters × 8, signal_length/8]. It then employs three upsampling modules to gradually reconstruct the feature map back to the original signal length. Each upsampling module consists of a transposed convolution layer, a feature concatenation operation, and a residual-attention block.

Transposed convolution, essentially a convolution operation in reverse, inserts zero-padding between elements of the input feature map and applies a convolution kernel to enlarge the feature map, restoring it to the spatial dimensions consistent with the corresponding encoder layer. The upsampled features are then concatenated with the output from the *x*-th encoder layer along the channel dimension, resulting in a composite feature map with doubled information. A subsequent 5×5 convolution compresses the number of channels back to base_filters × 4 (e.g., 256), followed by a residual block and an attention block to integrate the features and refine both the spatial and channel dimensions.

After passing through all three upsampling stages, a final layer with a 1×1 convolution maps the output to a single channel, yielding a denoised signal with dimensions [B, 1, L], matching the shape of the original input.

### 3.4. Evidential Regression Deep Network for Uncertainty Quantification

**Figure 4:**
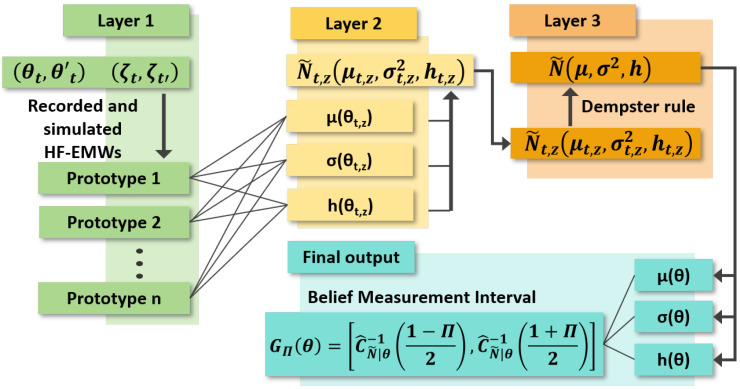
Architecture of evidential regression deep network.

This section proposes a DST-based evidential regression network to compute permittivity with the quantification of aleatory and epistemic uncertainty, as shown in Figure 4. The inputs of this network consist of an HF-EMW from real-world measurements of a concrete sample, an HF-EMW echo from an FDTD model that simulates the real-world measurement, and a denoised HF-EMW from the U-net-based model. The outputs are the permittivity of the concrete sample with the quantification of aleatory and epistemic uncertainty.

#### 3.4.1. Notation Definitions of Gaussian Random Fuzzy Number

The DST-based evidential regression network derives from the definition of a Gaussian random fuzzy number (GRFN) and generalized Dempster–Shafer theory (DST). A GRFN in the generalized DST can map x to a random variable *F*. Define the Gaussian fuzzy number (GFN) as a fuzzy subset of R with

(2) $$\mathrm{GFN}\left(x;m,h\right)=\exp\left(-\frac{h}{2}{\left(x-m\right)}^{2}\right),$$

where m∈R is the mode and h∈[0,+∞] is the precision. Two GFNs ${\mathrm{GFN}}_{1}\left(x;m_{1},h_{1}\right)$ and ${\mathrm{GFN}}_{2}\left(x;m_{2},h_{2}\right)$ can be combined as ${\mathrm{GFN}}_{12}\left(x;m_{12},h_{12}\right)$ with

(3) $$\begin{array}{c} m_{12}=\frac{h_{1}m_{1}+h_{2}m_{2}}{h_{1}+h_{2}},\;h_{12}=h_{1}+h_{2}. \end{array}$$

A GRFN can be expressed by a GFN with the mode of a Gaussian random variable. In detail, a Gaussian random variable with mean μ and variance $\sigma^{2}$ is formed as M:Ω→R in a probability space $\left(\Omega ,\sum_{\Omega },P\right)$. Then, a random fuzzy set $\tilde{N}:\Omega \to {\left[0,1\right]}^{R}$ in Equation (2) is written as

(4) $$\tilde{N}\left(\omega \right)=\mathrm{GFN}\left(M\left(\omega \right),h\right),$$

where $\tilde{N}\left(\mu ,\sigma^{2},h\right)$, called the GRFN, has a location parameter μ and two uncertainty parameters $\sigma^{2}$ and *h*. The two uncertainty parameters represent possibility and probability. In particular, a GRFN $\tilde{N}$ with h=+∞ equals to a Gaussian random variable with mean μ and variance $\sigma^{2}$. In contrast, the case with h=0 has $\tilde{N}\left(\omega \right)\left(x\right)=1$ for all ω∈Ω and x∈R. In addition, $\tilde{N}$ with $\sigma^{2}=0$ has a constant random variable *M* taking value μ, which can be seen as a possibilistic variable with possibility distribution GFN(μ,h).

Given two GRFNs ${\tilde{N}}_{1}\left(\mu_{1},\sigma_{1}^{2},h_{1}\right)$ and ${\tilde{N}}_{2}\left(\mu_{2},\sigma_{2}^{2},h_{2}\right)$, ${\tilde{N}}_{1}⊞{\tilde{N}}_{2}∼{\tilde{N}}_{1,2}\left(\mu_{12},\sigma_{12}^{2},h_{12}\right)$ can be defined based on the property of GFN in Equation (3) as

(5a) $$\mu_{12}=\frac{h_{1}\mu_{1}+h_{2}\mu_{2}}{h_{1}+h_{2}}$$

(5b) $$\sigma_{12}^{2}=\frac{h_{1}^{2}\sigma_{1}^{2}+h_{2}^{2}\sigma_{2}^{2}}{{\left(h_{1}+h_{2}\right)}^{2}}$$

(5c) $$h_{12}=h_{1}+h_{2}.$$

Obviously, the property is commutative and associative. Thus, two or more GRFNs can be aggregated by the accumulation operation, as shown in Equation (5).

#### 3.4.2. DST-Based Evidential Regression Network

This study extends the DST-based model in [31] to map the input into the permittivity value with the quantification of two types of uncertainty. The basic idea is to covert the input x, the combined tensor of the recorded and simulated HF-EMWs, into a Gaussian random fuzzy number (GRFN) via representation fusion, where μ(x), σ(x), and h(x) are the most plausible permittivity value and the aleatory uncertainty and epistemic uncertainty variability around this value, respectively. A permittivity computation with low uncertainty has μ(x)≈F, σ(x)≈0, and h(x)≈1. An intuitive description of the GRFN concept is provided in Appendix A. Given an input $x\in R^{p}$, the proposed DST-based evidential regression network can be described as the four steps shown in Figure 4:

**Step 1.**  The similarity between subset x and a *prototype* vector in the DST-based network is computed as
  (6) $$d_{z}\left(x\right)=\exp(-\gamma_{z}^{2}||x-\varsigma_{z}{\left|\right|}^{2}),$$
  where $\gamma_{z}$ is a scale associated with the prototype vector $\varsigma_{z}$; a continuous DST-based model has *Z* trainable prototype vectors with the *p* dimension, notated as $\varsigma_{1},\ldots \varsigma_{Z}$.
**Step 2.**  The similarity of $\varsigma_{z}$ is utilized to compute a GRFN as
  (7) $${\tilde{F}}_{z}\left(x\right)∼\tilde{N}\left(\mu_{z}\left(x\right),\sigma_{z}^{2},d_{z}\left(x\right)h_{z}\right)$$
  where μ, $\sigma^{2}$, and *h* are the mean, variance, and precision of GRFN $\tilde{N}\left(\mu ,\sigma^{2},h\right)$; the mean $\mu_{z}\left(x\right)$ is defined as
  (8) $$\mu_{z}\left(x\right)=\vartheta_{z}^{T}x+\vartheta_{z0}$$
  where $\vartheta_{z}$ and $\vartheta_{z0}$ are a trainable parameter vector and scale associated with prototype $\varsigma_{z}$; $\mu_{z}\left(x\right)$ is a prediction of material permittivity based on the inputs x, while $\sigma_{z}$ is the variation in the prediction $\mu_{z}\left(x\right)$. When the value of $||x-\varsigma_{z}\left|\right|$ tends toward 0, the precision $d_{z}\left(x\right)h_{z}$ is close to infinity, and ${\tilde{F}}_{z}\left(x\right)$ can highly support the hypothesis that $\mu_{z}\left(x\right)$ is the true value.
**Step 3.**  The *Z* GRFNs from prototypes $\varsigma_{1},\ldots \varsigma_{Z}$ are combined using a generalized Dempster rule operation ⊞ as $widetildeN(\mu \left(x\right),\sigma^{2},d\left(x\right)h)$ such that
  (9a) $$\mu \left(x\right)=\frac{\sum_{z=1}^{Z}d_{z}\left(x\right)h_{z}\mu_{z}\left(x\right)}{\sum_{z=1}^{Z}d_{z}\left(x\right)h_{z}},$$
  (9b) $$\sigma^{2}\left(x\right)=\frac{\sum_{z=1}^{Z}d_{z}^{2}\left(x\right)h_{z}^{2}\sigma_{z}^{2}\left(x\right)}{{\left(\sum_{z=1}^{Z}d_{z}\left(x\right)h_{z}\right)}^{2}},$$
  (9c) $$h\left(x\right)=\sum_{z=1}^{Z}d_{z}\left(x\right)h_{z}.$$
**Step 4.**  The aggregated GRFN $\tilde{N}\left(\mu ,\sigma^{2},h\right)$ can be transformed into a belief measurement interval (BMI) at a confidence level Π as
  (9d) $$G_{\Pi }\left(\theta \right)=\left[{\hat{C}}_{\tilde{N}|\theta }^{-1}\left(\frac{1-\Pi }{2}\right),{\hat{C}}_{\tilde{N}|\theta }^{-1}\left(\frac{1+\Pi }{2}\right)\right]$$
  where ${\hat{C}}_{\tilde{N}|\theta }^{-1}\left(\Pi \right)$ is the inverse of the cumulative distribution function ${\hat{C}}_{\tilde{N}|\theta }\left(\Pi \right)$, revealing how $\tilde{N}|\theta$ makes ${\hat{C}}_{\tilde{N}|\theta }$ return a value of Π. The inverse of the cumulative distribution function can be computed as
  (9e) $$\forall \;\Pi \in \left(0,1\right),\;P_{\theta ,\tilde{N}}\left(N\in G_{\Pi }\left(\theta \right)\right)\approx \Pi .$$
  For any Π∈(0,1], the Π-level interval in Equation (9d) can be regarded as a measurement at level Π. The BMI is the predicted permittivity range with a confidence level of Π. Thus, a BMI is a simple form of GFRN, allowing readers without a statistical background to understand it easily.

## 4. Results and Discussion

### 4.1. Experimental Details

**(1) Dataset**

Table 4 shows a dataset for demonstrating the effectiveness of the proposed method. A total of 300 concrete samples are made, for which their sizes are described in Section 3.2. All samples are scanned using ground-penetrating radars with antenna frequencies of 800 MHz, 1.0 GHz, 1.2 GHz, 1.6 GHz, and 2.0 GHz. In addition, the propagation of the HF-EMWs from the ground-penetrating radars is also simulated by the FDTD models in Section 3.2.

Each couple of recorded and simulated HF-EMWs is defined as an input for the experiment, while the true permittivity of the concrete sample is used as a label. Table 5 shows the volume fractions and permittivity of multiple phases in the four materials for FDTD simulation. The dataset in the experiment includes 1500 groups of the recorded HF-EMW, simulated HF-EMW, and true permittivity. The dataset’s split protocol is shown in Table 4.

**(2) Implementation details**

The proposed method is trained on a workstation equipped with two NVIDIA A6000 GPUs and 256 GB of RAM. Training is conducted for 40 epochs on the merged dataset using the ADAM optimizer, with a batch size of 16. The initial learning rate is set to 0.0001 and decays by a factor of 0.8 every 10 epochs. To reduce computational overhead, the encoder–decoder module is initialized with pre-trained weights from [32], while the ET-based regression module is trained from scratch. The complete training process takes approximately three hours on the specified hardware.

For HF-EMW denoising, the proposed method was compared with wavelet transform (WT), DnCNN [33], FFDNet [34], and REDNet [35]. All methods, except for the WT, are trained by the dataset in Table 4, where the input and label of a sample are the real-world recorded HF-EMWs and the FDTD simulation HF-EMWs, respectively.

For permittivity measurements with uncertainty quantification, the proposed model method was compared with a common two-layer neural network, LSTM [36], deep encoder [37], wave-processing transformer [38], swin transformer [39], SCUNet [32], and MCA-SCUNet [40]. All methods are trained by the dataset in Table 4. The input of a sample consists of a couple of recorded and simulated HF-EMWs, while the label indicates true permittivity.

**(3) Metrics**

A signal-to-noise ratio (SNR) and structural similarity index measure (SSIM) are adopted to evaluate the performance of HF-EMW denoising. The metrics SNR [41] and SSIM [42] are, respectively, defined as

(10a) $$SNR\left(\theta_{t}^{c},\theta_{t}^{\prime }\right)=\frac{1}{T}\sum_{t=1}^{T}10\log_{10}\left(\frac{\left|\right|\theta_{t}^{c}{\left|\right|}_{2}^{2}}{\left|\right|\theta_{t}^{c}-\theta_{t}^{\prime }{\left|\right|}_{2}^{2}}\right),$$

(10b) $$SSIM\left(\theta_{t}^{c},\theta_{t}^{\prime }\right)=\frac{1}{T}\sum_{t=1}^{T}\frac{\left(2\mu \left(\theta_{t}^{c}\right)\mu \left(\theta_{t}^{\prime }\right)+C_{1}\right)\left(2\sigma \left(\theta_{t}^{c},\theta_{t}^{\prime }\right)+C_{2}\right)}{\left(\mu {\left(\theta_{t}^{c}\right)}^{2}+\mu {\left(\theta_{t}^{\prime }\right)}^{2}+C_{1}\right)\left(\sigma^{2}\left(\theta_{t}^{c}\right)+\sigma^{2}\left(\theta_{t}^{\prime }\right)+C_{2})\right)},$$

where $\theta_{t}^{c}$ and $\theta_{t}$ are the *t*-th raw and denosied wave, respectively; μ, σ, and $\sigma^{2}$ are the mean, variance, and covariance of an HF-EMW, respectively.

The mean square error MSE and permittivity utility (PU) are used in the performance evaluation of the permittivity measurement with uncertainty quantification:

(11a) $$MSE\left(\theta \right)=\frac{1}{\left|D\right|}\sqrt{\frac{\sum_{\theta \in D}{\left(\varepsilon^{*}-\mu \left(\theta \right)\right)}^{2}}{\sum_{\theta \in D}{\left(\varepsilon^{*}-\bar{\mu }\right)}^{2}}}$$

(11b) $$PU=\left\{\begin{array}{cc} 0 & \mathrm{if}\;\varepsilon^{*}\notin G_{\Pi }\left(\theta \right)\\ 1-\frac{|\varepsilon^{*}-\mu \left(\theta \right)|}{{\hat{C}}_{\tilde{N}|\theta }^{-1}\left(\frac{1+\Pi }{2}\right)-\mu \left(\theta \right)+\alpha_{s}} & \mathrm{if}\;\varepsilon^{*}\in G_{\Pi }\left(\theta \right) \end{array}\right.$$

where $\varepsilon^{*}$ and μ(θ) are the labeled and predicted permittivity using HF-EMW θ; $\bar{\mu }$ is the average of all predictions in dataset *D*; $\alpha_{s}$ is a very small constant (=$10^{-8}$) used to prevent the denominator from being 0.

### 4.2. HF-EMW Echoes from an FDTD Model

A combined transmitting and receiving antenna was positioned above the model to perform simulated inspections at different frequencies. Five scan lines were sequentially arranged above the heterogeneous surface and base layer models to position the antenna. After filtering out the direct wave, the electric field intensity along the Z-direction was plotted, showing the radar signal waveforms propagating through each structural layer at various frequencies.

**Figure 5:**
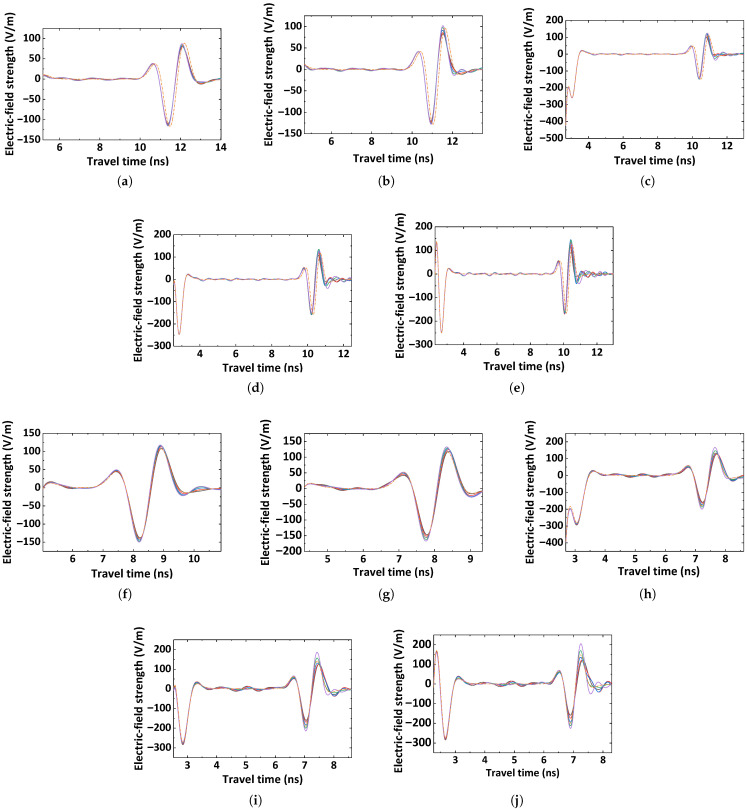
HF-EMW echoes from FDTD models: (**a**–**e**) are the HF-EMWs with frequencies 800 MHz, 1.0 GHz, 1.2 GHz, 1.6 GHz, and 2.0 GHz from a concrete sample of Material 1, and (**f**–**j**) are the HF-EMWs with frequencies 800 MHz, 1.0 GHz, 1.2 GHz, 1.6 GHz, and 2.0 GHz from a concrete sample of Material 3. Five solid curves are the simulated waves, and dotted curves are the recorded waves.

Figure 5 shows the electric field intensity curves in the Z-direction during radar wave propagation within the cement-stabilized gravel base layer under different excitation frequencies. HF-EMWs generated by a mid-frequency antenna exhibit waveforms that closely resemble those within the concrete sample. Clear reflections from the lower interface of the base layer, as well as partial reflections from the upper interface, can be distinctly identified.

Though the simulation and measurement results are similar, as shown in Figure 5, the peaks and troughs of the two types of HF-EMWs still have some differences, which mainly derive from the limited knowledge of the propagation of HF-EMWs. For example, the FDTD model has some assumptions, such as the air-layer configuration and permittivity in Section 3.2.2. Such limited knowledge inevitably leads to epistemic uncertainty in permittivity measurement. Therefore, the use of simulated and measured HF-EMWs as inputs allows the proposed method to quantify the epistemic uncertainty of permittivity measurement, as demonstrated in Section 4.2.

### 4.3. Denoising Results and Discussion

Table 6 shows the comparison results of the proposed method and other state-of-the-art (SOTA) methods on the denoising task. The proposed method achieves optimal results in terms of SNR and SSIM, indicating that it outperforms others on the denoising task. This demonstrates that the proposed method is more suitable than others for removing measurement noise, such as randomly distributed cement and aggregates in cement-based composites. This property enables the proposed approach to quantify aleatory uncertainty, which will be demonstrated in the next section.

**Figure 6:**
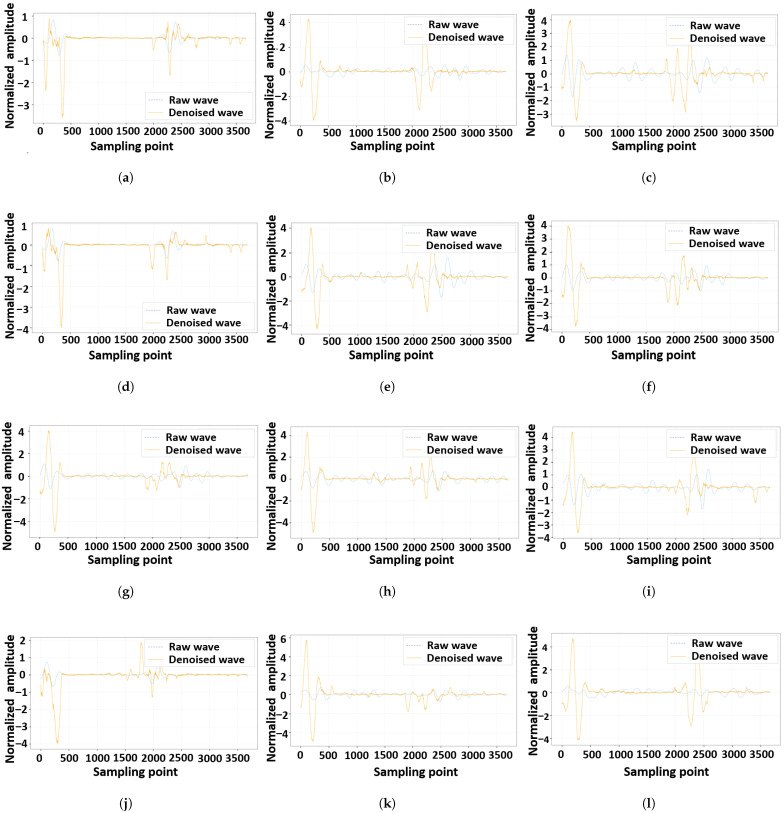
Denosing results of the proposed method. (**a**) Example 1, (**b**) Example 2, (**c**) Example 3, (**d**) Example 4, (**e**) Example 5, (**f**) Example 6, (**g**) Example 7, (**h**) Example 8, (**i**) Example 9, (**j**) Example 10, (**k**) Example 11, and (**l**) Example 12.

Figure 6 provides some HF-EMWs before and after denoising. In general, the proposed method can retain the shapes of two pairs of peaks and troughs in the HF-EMWs and remove noise waves within the ranges of these two pairs. The two pairs of peaks and troughs in an HF-EMW originate from the incidence and emergence of the wave in a concrete sample, while the noise waves derive from the wavelets owing to randomly distributed cement and aggregates. The difference between the raw and denoised HF-EMWs characterizes the effects of the measurement noises. Therefore, the denoising deep network provides a potential way to quantify the aleatory uncertainty in the proposed method, which will be proven in the next section.

### 4.4. Permittivity Characterization with Uncertainty Quantification

Table 7 shows the MSE of the proposed method and other SOTAs. In general, the proposed one exceeds the others in the Bayesian probability framework in terms of MSE. This indicates that the proposed method can accurately predict the permittivity of cement-based samples with minimal error. This mainly benefits from the fact that the proposed method can quantify the epistemic uncertainty of permittivity measurement by comparing simulated and recorded HF-EMWs and characterize the aleatory uncertainty by comparing the HF-EMWs before and after denoising. However, the other network in the Bayesian probability framework, such as MCA-SCUNet and swin transformer only captures the randomness aspect of the data, but neither ambiguity nor incompleteness is captured, which is the inherent epistemic uncertainty in HF-EMWs.

**Figure 7:**
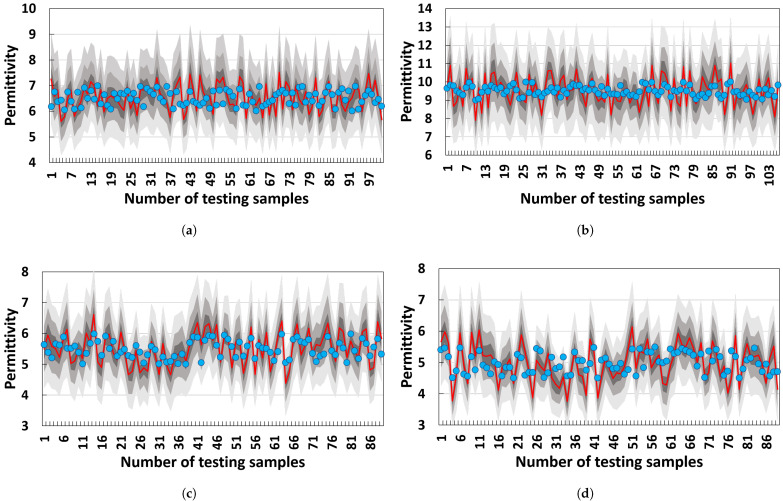
Uncertainty quantification of permittivity measurement using belief measurement intervals: (**a**) Material 1, (**b**) Material 2, (**c**) Material 3, and (**d**) Material 4. The red lines are the labeled permittivity, while the blue points are the predicted one. The three grays are the BMIs with Π in [0.5, 0.05, 0.99].

**Figure 8:**
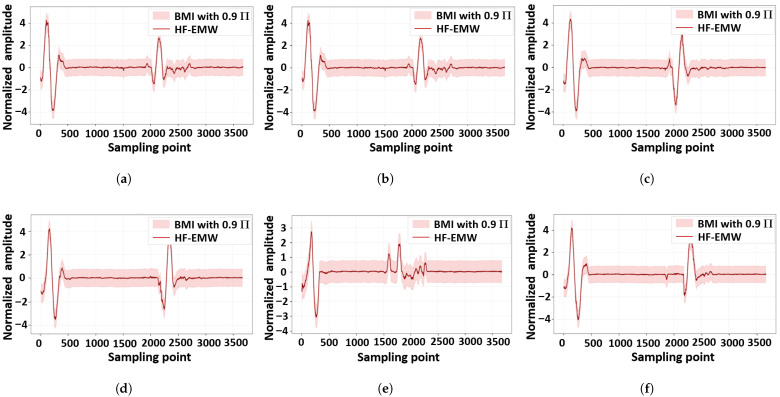
Recorded high-frequency electromagnetic waves with uncertainty quantification using belief measurement intervals. (**a**) Example 1, (**b**) Example 2, (**c**) Example 3, (**d**) Example 4, (**e**) Example 5, (**f**) Example 6.

Table 7 also shows the PU of the proposed method and other SOTAs. The proposed one outperforms the others in PU terms. This indicates that the proposed method can accurately quantify the uncertainty of permittivity measurements. Figure 7 shows a visual explanation of this advantage via BMIs. In general, the predicted permittivity of the proposed method mainly falls into the BMI with a Π of 0.95, demonstrating that the permittivity measurement of the proposed method can quantify the two types of uncertainty and reduce their negative effects on the final measurement. In detail, Figure 8 shows the BMIs of HF-EMWs. A large range of BMI in an HF-EMW mainly falls into the 100–600 sampling points, where the HF-EMWs propagate in the samples. This propagation always leads to epistemic uncertainty due to the limited knowledge of real wave-propagation paths and aleatory uncertainty due to multi-phase heterogeneous materials.

**Figure 9:**
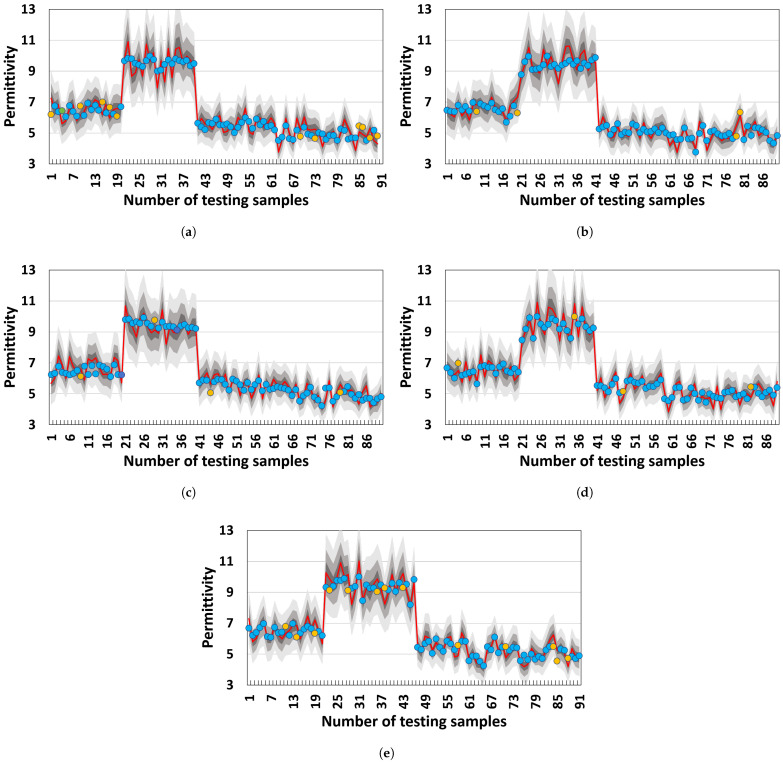
Uncertainty quantification of permittivity measurement using belief measurement intervals: (**a**) 800 MHz, (**b**) 1.0 GHz, (**c**) 1.2 GHz, (**d**) 1.6 GHz, and (**e**) 2.0 GHz. The predicted results do not fall into the BIM, with Π=0.99 highlighted in yellow. The red lines are the labeled permittivity, while the blue points are the predicted one. The three grays are the BMIs with Π in [0.5, 0.05, 0.99].

Figure 9 shows the uncertainty quantification under various HF-EMW frequencies. The measurement results for 1.0 GHz, 1.2 GHz, and 1.6 GHz all fall within the BMI of 0.95, whereas those for 800 MHz and 2.0 GHz do not. This phenomenon indicates that the HF-EMWs with 800 MHz and 2.0 GHz frequencies have higher uncertainty than those with 1.0 GHz, 1.2 GHz, and 1.6 GHz frequencies. Theoretically, an HF-EMW with a low frequency has a low resolution that always leads to aleatory uncertainty, which is also known as a measurement error. An HF-EMW with high frequencies has a high resolution and a small wavelength. Although higher resolutions have low aleatory uncertainty, a small wavelength always leads to Fresnel diffraction in multi-phase heterogeneous concrete, which generates sub-waves in the HF-EMW propagation path and leads to epistemic uncertainty.

Table 8 shows the ablation study of the denosing and FDTD simulation. Once the denoising deep network is removed (Model 2), the MSE and PU of the proposed method decrease. This is mainly because the proposed method cannot quantify the aleatory uncertainty due to the measurement noise and multi-phase heterogeneous materials. Therefore, a reasonable denoising model is crucial for permittivity measurements using HF-EMWs.

Table 8 also indicates that the MSE and PU of the proposed method decrease if we do not use the simulated HF-EMW as an input (Model 3). This is because the proposed method cannot capture the knowledge of HF-EMW propagation paths without the simulated HF-EMW. Therefore, an FDTD simulation of HF-EMW propagation offers a new approach to enhancing the accuracy of permittivity measurements by providing the necessary knowledge.

Table 8 also indicates that the MSE and PU of the proposed method decrease if we do not use the DST-based regression network as the final output (Model 4). This is because the DST-based regression network quantifies the two types of uncertainties in the permittivity measurement, which represent and reduce the negative effect of uncertainty. Therefore, a DST-based regression network is necessary for the permittivity measurement with uncertainty quantification.

## 5. Conclusions

This study proposes a DST-based evidential regression deep network for conducting permittivity measurements with uncertainty quantification. This method first proposes an FDTD model with multi-phase heterogeneous concrete materials to simulate the HF-EMW propagation in a concrete sample or structure, obtaining the HF-EMW echo. A U-net-based model is then proposed to denoise an HF-EMW. Finally, a DST-based evidential regression network is proposed to compute the permittivity with aleatory and epistemic uncertainty using a Gaussian random fuzzy number (GRFN). An experiment involving 1500 samples is conducted to demonstrate the effectiveness of the proposed method. The following conclusions can be drawn:

1. The proposed method measures the permittivity of four types of multi-phase heterogeneous concrete materials with an MSE of 7.50%, exceeding other SOTA methods in this task. The proposed method is also stable with respect to the four concrete types. Therefore, a user can directly use the method to measure the permittivity of concrete materials using recorded HF-EMWs.
2. The proposed method can quantify the uncertainty in permittivity measurement using a GRFN-based BMI. The predicted permittivity mainly falls into the BMI with a 0.95 Π, demonstrating that the proposed method can quantify and remove the effects of aleatory and epistemic uncertainty in the permittivity measurement.
3. The superiority of the proposed method benefits from the FDTD simulation and the denoising deep network. The FDTD simulation quantifies epistemic uncertainty due to limited knowledge, such as HF-EMW propagation. The denoising deep network quantifies aleatory uncertainty due to the measurement’s noise, such as the multi-phase heterogeneous property of concrete materials.
4. The applicability of the proposed method is limited in cement concrete and cement-stabilized macadam. The next step is to extend the proposed method to other concrete materials, such as fiber-reinforced concrete and lightweight concrete, via conducting an experiment similar to the one outlined in Section 4.1. In the practical implementation, the proposed DST-based deep network can be embedded into a GPR with edge computing capabilities, and engineers can rapidly assess material qualities during construction or inspection.

## Figures and Tables

**Table 1:** Comparison of permittivity measurement methods.

Method	Frequency Range	Contact Type	Advantages	Limitations
Coaxial Probe	MHz–GHz	Contact	Simple setup, broadband	Limited to flat surfaces, sensitive to pressure
Waveguide	GHz	Contact	High accuracy	Requires machining, limited bandwidth
Free-space Measurement	GHz–THz	Non-contact	Non-destructive, broadband	Sensitive to alignment, needs calibration
Resonant Cavity	Discrete GHz	Contact	Extremely high accuracy	Narrowband, complex sample prep
Time-Domain Reflectometry	MHz–GHz	Contact	Time-resolved info, moderate complexity	Limited resolution at high frequencies
THz Time-Domain Spectroscopy	0.1–10 THz	Non-contact	Ultra-high resolution, molecular sensitivity	Expensive, semi-transparent samples only

**Table 2:** Mass proportions of aggregates in different concrete materials.

Size/mm	0.6–2.36	2.36–4.75	4.75–9.5	9.5–16	16–19	19–26.5	26.5–37.5
Cement concrete (Material 1)	22.80	7.67	10.86	10.67	12.44	17.00	18.56
Cement concrete (Material 2)	20.62	8.79	11.32	12.78	9.45	16.14	20.90
Cement-stabilized macadam (Material 3)	25.92	16.30	20.00	11.11	8.89	17.78	/
Cement-stabilized macadam (Material 4)	22.41	17.24	21.32	14.32	7.82	16.89	/

**Table 3:** Mass proportions of aggregates in different pavement materials.

Parameter	Value	Parameter	Value
Model Size (m)	1.5×0.6/0.4×0.00044	Antenna Step (m)	0.01
Grid Step (m)	0.00044×0.00044×0.00044	Scan Lines	146
Time Window (ns)	16/12	Excitation source frequency (GHz)	0.8, 1.0, 1.2, 1.6, and 2.0 GHz

**Table 4:** Detailed information on the experimental dataset.

	Number of Samples	Training	Validation	Testing
Materials 1	67	193	41	101
Materials 2	75	231	38	106
Materials 3	68	214	37	89
Materials 4	90	262	34	154
Total	300	900	150	450

**Table 5:** Volume fraction and permittivity of four concrete materials.

	Component	Material 1	Material 2	Material 3	Material 4
Volume fraction	Coarse aggregate	72.17	72.17	76.98	76.98
	Fine aggregate	4.82	4.82	14.25	14.25
	Cement mortar	22.01	22.01	3.76	3.76
	Pore	1.00	1.00	5.00	5.00
Permittivity	Aggregate	8.00	8.00	10.00	10.00
	Cement	10.30	23.34	9.69	18.42
	Average in (1)	8.62	11.46	9.93	9.86

**Table 6:** Performance of HF-EMW denoising.

	Train_Loss	Train_ACC	SNR (dB)			SSIM		
WT	0.3648	0.8344	20.89	20.97	20.78	0.7315	0.7330	0.7199
DnCNN	0.5175	0.7101	22.54	21.81	22.27	0.7821	0.7553	0.7498
FFDNet	0.5979	0.6535	24.12	24.05	23.98	0.8234	0.8219	0.8102
REDNet	0.3803	0.8106	26.45	26.38	26.52	0.8776	0.8761	0.8283
ours	**0.0740**	**0.9312**	**29.71**	**29.65**	**29.78**	**0.9287**	**0.9279**	**0.9295**

The bolds are the best results in the testing set.

**Table 7:** Testing results of permittivity FL measurements.

Methods	MSE/%					PU/%				
	Overall	Materials 1	Materials 2	Materials 3	Materials 4	Overall	Materials 1	Materials 2	Materials 3	Materials 4
ANN	17.34	15.53	16.78	17.39	19.65	54.14	51.37	56.6	55.05	53.54
LSTM	13.5	14.36	14.85	14.2	10.57	54.06	53.05	51.35	55.51	56.31
Deep encoder	14.27	12.56	16.93	15.26	12.31	58.79	56.52	59.2	58.33	61.1
Wave-processing transformer	12.04	11.09	7.91	12.71	16.45	64.96	65.44	62.6	62.33	69.47
Swin transformer	11.03	7.98	10.01	9.3	16.83	66.29	69.71	64.21	64.7	66.54
SCUNet	11.09	10.18	10.19	11.07	12.9	70.19	68.67	71.25	69.38	71.47
MCA-SCUNet	9.08	8.08	7.75	10.47	10	72.87	70.5	75.14	74.12	71.72
Ours	7.5	6.85	6.48	8.01	8.64	74.7	74.09	75.34	76.3	73.06

**Table 8:** Ablation study of the proposed method.

	Denoise	FDTD Simulation	DST-Based Regression	MSE/%	PU/%
Model 1	✓	✓	✓	7.50	74.70
Model 2	-	✓	✓	10.08	64.62
Model 3	✓	-	✓	9.34	68.72
Model 4	✓	✓	-	9.14	61.73
Model 5	-	-	✓	10.45	64.36
Model 6	✓	-	-	10.08	63.88

**Figure A1:**
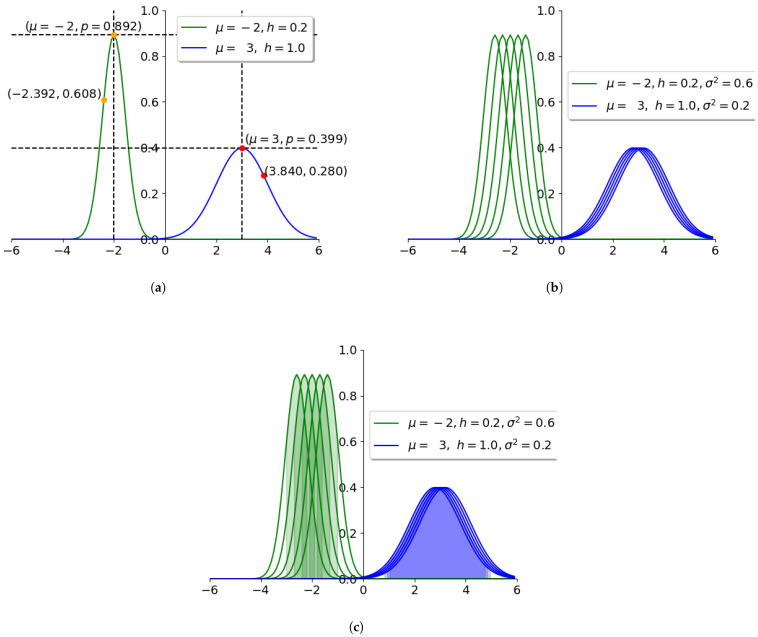
Two visual examples of (**a**) Gaussian fuzzy numbers, (**b**) Gaussian random fuzzy numbers, and (**c**) BMIs.

## Data Availability

The original contributions presented in this study are included in the article. Further inquiries can be directed to the corresponding author.

## Appendix A

The concept of a GRFN derives from the Gaussian fuzzy number (GFN) with mode μ and variance σ, notated as GFN(μ,σ), which is a fuzzy subset of R with membership function $\theta \to \exp\left(-\frac{\sigma }{2}{\left(x-\mu \right)}^{2}\right)$. Each point in the membership function of a GFN indicates the probability that the selected value is the truth. The value μ can be the predicted permittivity in the proposed method, while the variance σ is the aleatory uncertainty. Figure A1a shows two examples of GFNs. For the green one, GFN(−2,0.2) indicates that the correct probability is 0.892 if one selects −2 as the final prediction, while the probability is 0.608 with a final prediction of −2.392. A GFN presents the aleatory uncertainty of a prediction with respect to μ, where true permittivity is around μ, but one cannot determine a precise value due to the random error, such as the multi-phase heterogeneous property of a cement-based composite.

The epistemic uncertainty is then introduced by converting GFN into a Gaussian random fuzzy number (GRFN) with mean μ, variance $\sigma^{2}$, and precision *h*, notated as $\tilde{N}\left(\mu ,\sigma ,h\right)$. A GRFN is a GFN for which its mode is also a GFN, $N(\mu ,\sigma^{2})$, indicating that the mode of the GFN includes epistemic uncertainty owing to the knowledge limitation of permittivity measurements. Figure A1b extends the two examples in Figure A1a into GRFNs. In summary, μ, σ, and *h* in a GRFN is the predicted permittivity value, the quantification of aleatory uncertainty, and the quantification of epistemic uncertainty, respectively.

Finally, a BMI of a GRFN is the long-run prediction of the confidence level Π such that the true value theoretically falls in the interval with aleatory and epistemic uncertainty, as shown in Figure A1c. A BMI is a general form of the classic statistical form μ±h in a property measurement. The classic form μ±h:=[μ−h,μ+h] represents a measurement mean μ with uncertainty *h*, where the truth is around μ but cannot be used to determine a precise value. However, the classic form cannot show that the probability decreases with a distance increase in the mean. A BIM is equivalent to the classic representation when $\sigma^{2}=0$ and h=+∞.
